# Neuronal human *BACE1* knockin induces systemic diabetes in mice

**DOI:** 10.1007/s00125-016-3960-1

**Published:** 2016-05-02

**Authors:** Kaja Plucińska, Ruta Dekeryte, David Koss, Kirsty Shearer, Nimesh Mody, Phillip D. Whitfield, Mary K. Doherty, Marco Mingarelli, Andy Welch, Gernot Riedel, Mirela Delibegovic, Bettina Platt

**Affiliations:** School of Medical Sciences, College of Life Sciences and Medicine, University of Aberdeen, Institute of Medical Sciences, Foresterhill, Aberdeen, AB25 2ZD Scotland UK; Department of Diabetes and Cardiovascular Science, Centre for Health Science, University of the Highlands and Islands, Inverness, Scotland UK

**Keywords:** ^18^FDG-PET, Ceramide, ER stress, Glucose homeostasis, Insulin signalling, Neuronal BACE1

## Abstract

**Aims:**

β-Secretase 1 (BACE1) is a key enzyme in Alzheimer’s disease pathogenesis that catalyses the amyloidogenic cleavage of amyloid precursor protein (APP). Recently, global *Bace1* deletion was shown to protect against diet-induced obesity and diabetes, suggesting that BACE1 is a potential regulator of glucose homeostasis. Here, we investigated whether increased neuronal BACE1 is sufficient to alter systemic glucose metabolism, using a neuron-specific human *BACE1* knockin mouse model (PLB4).

**Methods:**

Glucose homeostasis and adiposity were determined by glucose tolerance tests and EchoMRI, lipid species were measured by quantitative lipidomics, and biochemical and molecular alterations were assessed by western blotting, quantitative PCR and ELISAs. Glucose uptake in the brain and upper body was measured via ^18^FDG-PET imaging.

**Results:**

Physiological and molecular analyses demonstrated that centrally expressed human BACE1 induced systemic glucose intolerance in mice from 4 months of age onward, alongside a fatty liver phenotype and impaired hepatic glycogen storage. This diabetic phenotype was associated with hypothalamic pathology, i.e. deregulation of the melanocortin system, and advanced endoplasmic reticulum (ER) stress indicated by elevated central C/EBP homologous protein (CHOP) signalling and hyperphosphorylation of its regulator eukaryotic translation initiation factor 2α (eIF2α). In vivo ^18^FDG-PET imaging further confirmed brain glucose hypometabolism in these mice; this corresponded with altered neuronal insulin-related signalling, enhanced protein tyrosine phosphatase 1B (PTP1B) and retinol-binding protein 4 (RBP4) levels, along with upregulation of the ribosomal protein and lipid translation machinery. Increased forebrain and plasma lipid accumulation (i.e. ceramides, triacylglycerols, phospholipids) was identified via lipidomics analysis.

**Conclusions/interpretation:**

Our data reveal that neuronal BACE1 is a key regulator of metabolic homeostasis and provide a potential mechanism for the high prevalence of metabolic disturbance in Alzheimer’s disease.

**Electronic supplementary material:**

The online version of this article (doi:10.1007/s00125-016-3960-1) contains peer-reviewed but unedited supplementary material, which is available to authorised users.

## Introduction

The incidence of type 2 diabetes and Alzheimer’s disease is rising at an alarming rate. Type 2 diabetes increases the risk of Alzheimer’s disease [1]; however, systemic glucose intolerance and insulin resistance are also reported in dementia patients without a history of diabetes [2]. Alzheimer’s disease brains exhibit defective neuronal insulin signalling [3] and glucose hypometabolism in 2-[^18^F]fluoro-2-deoxy-d-glucose positron emission tomography (^18^FDG-PET) studies [4]. Further, mouse models of both diseases share similar cognitive phenotypes [5], and increased susceptibility to high-fat diet (HFD)-induced diabetes is observed in Alzheimer mouse models compared with controls [6]. Collectively, the overlapping pathology indicate common pathogenic factors, although their molecular foundations remain unclear.

β-Secretase 1 (BACE1) is implicated in Alzheimer’s disease as the enzyme responsible for the rate-limiting step in β-amyloid (Aβ) production [7]. Brain BACE1 levels increase with age [8], particularly in Alzheimer’s disease [9], and following pathological events [10, 11]. *Bace1* deletion abolishes Aβ deposition and rescues cognitive deficits in *APP* mutant mice [12]. We recently confirmed that neuron-specific knockin of human (*h*)*BACE1* induces Aβ accumulation, promotes brain inflammation and recapitulates Alzheimer’s disease-like phenotypes in mice in the absence of mutant *APP* expression [13], suggesting that BACE1 represents a molecular risk factor for sporadic Alzheimer’s disease.

Although studied principally for its role in amyloidosis, BACE1 has multiple substrates other than APP [14], comprising transmembrane proteins involved in intercellular signalling. BACE1 expression is predominantly neuronal, although *BACE1* mRNA is also found in the liver, skeletal muscle and pancreas [15], with pancreatic mRNA encoding an inactive isoform [7, 16]. A potential role for BACE1 in metabolic regulation has only recently emerged, as *Bace1* knockout improved glucose metabolism and protected mice from HFD-induced obesity and diabetes [17]. Conversely, the induction of insulin deficiency via systemic streptozotocin (STZ) injection raised central BACE1 levels [18], and this was associated with endoplasmic reticulum (ER) stress [19]. Thus, BACE1 may contribute to metabolic regulation; however, it remains to be established whether BACE1 mediates the association between type 2 diabetes and Alzheimer’s disease.

To elucidate the contribution of neuronal BACE1 to systemic glucose regulation and lipid metabolism, we characterised central and peripheral metabolic changes in brain-specific *hBACE1* (PLB4) knockin mice [13]. Because global deletion of *Bace1* protected mice from diet-induced diabetes [17], we hypothesised that neuronal BACE1 may regulate system metabolism in addition to inducing brain pathologies relevant to Alzheimer’s disease [13]. We provide evidence that BACE1-induced hypothalamic dysregulation causes systemic diabetes, which may explain the high comorbidity of diabetes and Alzheimer’s disease in ageing populations.

## Methods

### Animals

All animals were housed and tested in accordance with European (Directive on the Protection of Animals used for Scientific Purposes, 2010/63/EU) and UK Home Office regulations, experiments were approved by the University of Aberdeen Ethics Board and performed in accordance with the Animal (Scientific Procedures) Act 1986 and following Animal Research: Reporting of In Vivo Experiments (ARRIVE) guidelines. The transgenic PLB4 and WT lines were generated and bred as previously described [13], experimenters were not blinded to genotype. All mice were housed in single sex groups, unless food and water consumption was being measured. Physiological assessments were performed in male mice at 3, 4, 5 and 8 months of age. All mice were killed by neck dislocation. Five-hour-fasted mice aged 3 and 8 months were used for liver assays, and all postmortem molecular analyses of signalling pathways (western blotting and quantitative real-time PCR [qPCR]) were performed using samples from 8-month-old mice. Glucose-stimulated insulin secretion (GSIS) was determined in 4-month-old mice at 0, 15 and 30 min time points during glucose tolerance tests (GTTs). Blood leptin content was determined from baseline reading at 3, 4 and 8 months in a 5 h fasted state. EchoMRI scans (EchoMRI, Houston, TX, USA) were performed on mice at ages 4, 5 and 8 months. Additional 6-month-old mice were used for lipidomic analysis and ^18^FDG-PET imaging. Body weights were recorded and food and water consumption (in g) were measured for 2 weeks in 5- and 8-month-old male mice.

### Metabolic measurements

Tail blood glucose was determined using an AlphaTRAK glucometer (Berkshire, UK). GTTs were performed (as described in [6, 20]) in 5-h fasted male mice at age 3 (WT, *n* = 4; PLB4 *n* = 7), 4 (WT, *n* = 8; PLB4, *n* = 7), 5 (WT, *n* = 9; PLB4, *n* = 15) and 8 (WT, *n* = 4; PLB4 *n* = 4) months. Fasting blood glucose was determined at baseline (time 0) before i.p. injection of bolus glucose (20%; 2 g/kg body weight). Blood glucose clearance was assessed at 15, 30, 60 and 90 min post injection in 3-, 5- and 8-month-old mice; and at 0, 15 and 30 min post injection in 4-month-old mice.

### Serum immunoassays

Tail- or trunk-derived blood was collected from 5-h fasted mice aged 3 (WT, *n* = 6; PLB4, *n* = 8), 4 (WT, *n* = 7; PLB4, *n* = 8), 5 (WT, *n* = 9; PLB4, *n* = 12) and 8 (WT, *n* = 8; PLB4, *n* = 10) months into serum separator microtubes; serum was used for insulin and leptin determination (Insulin ELISA, Millipore, Darmstadt, Germany; leptin ELISA, CrystalChem, Zaandam, the Netherlands). A multiplex assay (customer-designed mouse metabolic hormone Magnetic Multiplex Assay; Merck Millipore, Darmstadt, Germany) was used for the simultaneous quantification of leptin, amylin (active form), C-peptide 2, glucose-dependent insulinotropic peptide (GIP; total), pancreatic polypeptide, peptide tyrosine tyrosine (PYY), IL-6 and resistin following the manufacturer’s instructions. Triacylglycerol and glycogen assays were used as previously described [20].

### Comparative lipidomics plasma and brain analyses

Frozen plasma and forebrain samples from 6-month-old male mice (WT, *n* = 8; PLB4, *n* = 8) were used for global lipid analysis using liquid chromatography–mass spectroscopy (LC–MS). Plasma lipids were extracted from samples according to the Folch method. Lipids were solvent extracted in methanol/chloroform (2:1 vol./vol.). The lipid extracts were subsequently analysed by LC–MS in positive and negative ion modes with a C18 column and a water/acetonitrile/isopropanol gradient using an Exactive Orbitrap system (Thermo Scientific, Hemel Hempstead, UK) . Lipidomic datasets were processed using Progenesis QI software (version 2.0, Non-Linear Dynamics, Newcastle upon Tyne, UK) and searched against LIPID MAPS (www.lipidmaps.org/) and the Human Metabolome Database (www.hmdb.ca/).

### Brain and upper body PET/CT imaging

Six-month-old female mice (WT, *n* = 10; PLB4, *n* = 9) were imaged using an Argus GE dual ring PET/CT scanner (Sedecal, Madrid, Spain) using our published protocol [21].

### Immunoblotting

All tissues were lysed in RIPA buffer [as described in [6]. Tissue from 5-h fasted mice was immunoblotted using rabbit polyclonal antibodies diluted 1:1000 in TRIS-buffered saline containing Tween-20, 5% BSA and 0.05% sodium azide. Antibodies raised against human BACE1 (C-terminal 485–501; no. 195111, Calbiochem, UK); phospho-protein kinase B (p-Akt_Ser473_; no. 4060), total Akt (Akt; no. 9272), phospho-glycogen synthase kinase-3β (p-GSK-3β_Ser21/9_; no. 9331), phospho-ribosomal protein S6 (p-rpS6_Ser235–236_; no. 4858), total rpS6 (rpS6; no. 2217), phospho-S6 kinase (p-S6K_Thr389_; no. 9234), total S6 kinase (S6K; no. 2708), phospho-mammalian target of rapamycin (p-mTOR_Ser2448_; no. 5536), mTOR (no. 2983), phospho-glycogen synthase (p-GS_Ser641_; no. 3891), phospho-eukaryotic translation initiation factor 2α (p-eIF2α_Ser51_; no. 3398), total eIF2α (no. 5324), protein kinase RNA-like ER kinase (PERK; no. 5683; all obtained from Cell Signaling Technology, Leiden, the Netherlands); insulin receptor β (IRβ; C-19, no. Sc-711; 1:500 dilution), apolipoprotein (ApoE; no. Sc-6384), mouse monoclonal protein tyrosine phosphatase 1B (PTP1B; no. Sc-14021), C/EBP homologous protein (CHOP; also known as GAD153, no. Sc-7351; Santa Cruz, Heidelberg, Germany); and retinol-binding protein 4 (RBP4; no. A0040, Dako, Glastrup, Denmark) were used following manufacturer’s instructions and as described before [6, 13]. Coomassie Blue (PhastGel Blue R; GE Healthcare, Watford, UK) was used as a loading control [13, 22].

### Gene expression

RNA was extracted from frozen hypothalamic and cortical samples using TRI Reagent, as previously described [23]. Target genes (*hBACE1* and mouse *Pomc*, *Mc4R*, *Npy*, *LepR*, *Ptp1b*, *Chop*, *Cd11* and *Cd68*) were amplified by qPCR using GoTaq Master Mix (Promega, Madison, WI, USA). The geometric mean of three commonly used reference mRNAs (*Ywhaz*, *Nono* and *Actb*) was used to normalise data.

### Statistical analysis

Prism 5 (GraphPad, La Jolla, CA, USA) was used for statistical analyses, and data are expressed as the means ± SEM. All molecular and genetic data were adjusted to loading controls or housekeeping genes and calculated relative to WT. Two-tailed *t* tests with Welch’s correction were used for unbiased comparisons between transgenic mice and controls. Group analyses used two-way ANOVA (with repeated measures where appropriate), followed by Bonferroni post hoc tests.

## Results

### Systemic glucose homeostasis is impaired in PLB4 mice

**Fig. 1:**
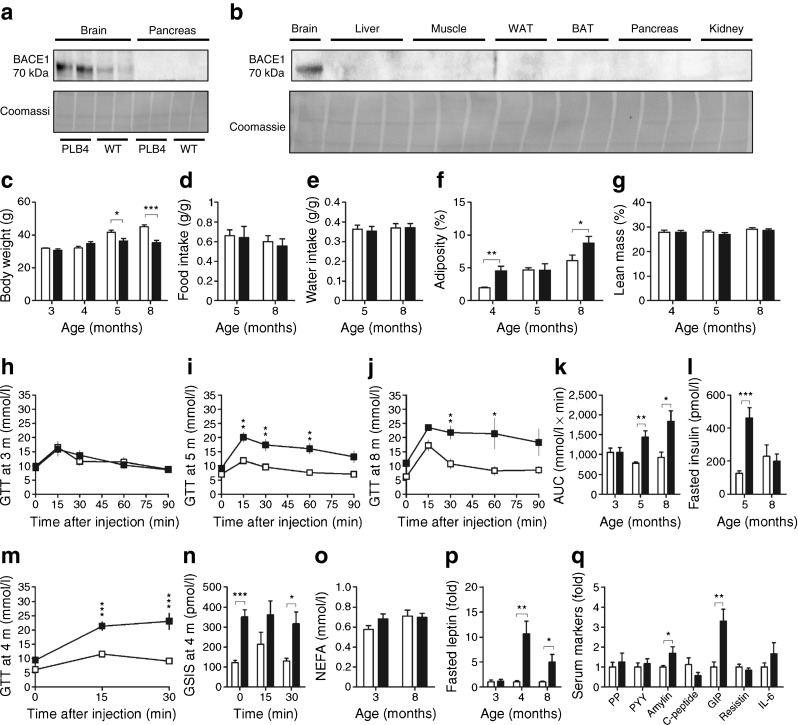
Systemic diabetes in neuronal *hBACE1* knockin mice. (**a**) BACE1 protein content in soluble lysates from neuronal and pancreatic tissues from PLB4 and WT mice. (**b**) BACE1 protein screening in other tissue types from PLB4 mice only. (**c**) Body weight of WT and PLB4 mice at 3, 4, 5 and 8 months (m) of age. (**d**, **e**) Normalised food (**d**) and water (**e**) intake in 5- and 8-month-old mice. (**f**, **g**) Body composition data obtain from EchoMRI scans showing adipose (**f**) and lean mass (**g**) in mice aged 4, 5 and 8 months. (**h**–**j**) GTTs at age 3 (*p* > 0.05), 5 (*p* < 0.01) and 8 (*p* < 0.05) months (m). (**k**) Total glucose excursions during GTTs. (**l**) Fasted serum insulin concentrations in 5- and 8-month-old PLB4 and WT mice. (**m**) GTTs in 4-month-old mice (*p* < 0.001; onset of defective glucose disposal). (**n**) GSIS in 4-month-old mice during GTTs. (**o**) Serum NEFA at 3 and 8 months of age. (**p**) Fasted serum leptin levels in PLB4 vs WT mice at 3, 4 and 8 months of age. (**q**) Serum markers detected in 8-month-old PLB4 mice using an enzymatic multiplex assay. White bars, WT mice; black bars, PLB4 mice. Data represent means + SEM or (**p**, **q**) means + SEM normalised to WT values. *p < 0.05, **p < 0.01, ***p < 0.001

We first confirmed that human BACE1 expression was brain specific (Fig. 1a,b) and then established that body weights were normal in PLB4 mice aged up to 4 months (Fig. 1c) but decreased compared with wild-type (WT) controls at 5 and 8 months. Food and water intake adjusted to body weight were not affected (Fig. 1d,e). Despite the lean phenotype, adipose tissue mass was mildly increased in PLB4 mice vs WT at 4 and 8 months of age (Fig. 1f), while lean mass was unaffected (Fig. 1g), as demonstrated by EchoMRI. Furthermore, 5-h fasted PLB4 mice exhibited elevated blood glucose levels, progressive severe glucose intolerance (Fig. 1h–k) and impaired GSIS from 4 months of age (Fig. 1m,n). Serum insulin levels were also raised (hyperinsulinaemia) at 5 months but were similar to WT levels at 8 months (Fig. 1l), despite continued hyperglycaemia. Serum NEFA were increased in 3-month-old PLB4 mice, although this was not statistically significant (*p* = 0.06), and were comparable between groups at 8 months of age (Fig. 1o).

### Altered plasma metabolic homeostasis and hyperleptinaemia

Levels of the adipocyte-derived hormone, leptin, which strongly correlate with obesity and diabetes, were initially normal in PLB4 mice, but were drastically elevated at 4 and 8 months (Fig. 1p) when adiposity was increased.

Since body mass and long-term energy expenditure are tightly regulated by complex signalling networks between the CNS and peripheral systems, we screened serum samples from 8-month-old PLB4 mice for markers of homeostatic control using a multiplex assay (Fig. 1q). Concentrations of fasting C-peptide were low, indicative of defective proinsulin synthesis, while amylin levels (co-secreted with insulin) were enhanced. GIP was also upregulated in PLB4 mice compared with controls.

### Impaired hepatic glycogen synthesis, insulin resistance and fatty liver phenotype

**Fig. 2:**
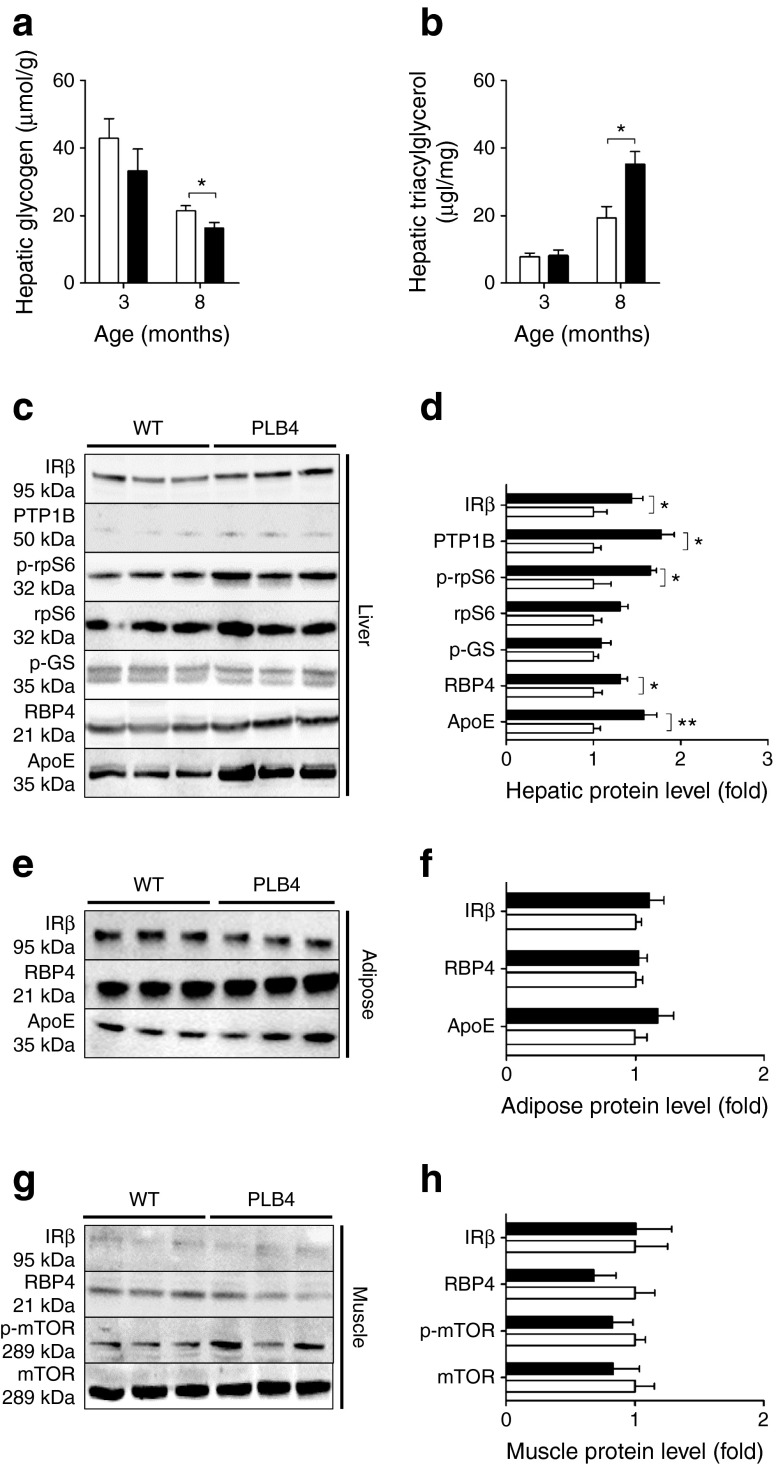
Hepatic pathology induced by brain-specific *hBACE1* knockin. (**a**) Hepatic glycogen content of 3- and 8-month-old PLB4 and control mice. (**b**) Triacylglycerol concentrations of liver tissue from 3- and 8-month-old PLB4 and WT mice. (**c**) Representative immunoblots showing insulin signalling related markers in liver tissue from 8-month-old WT and PLB4 mice, and (**d**) quantification of the relative protein content. (**e**) Representative IRβ, RBP4 and ApoE immunoblots of WAT, and (**f**) quantification of relative protein content. (**g**) Hindlimb skeletal muscle immunoblots and (**h**) quantification of relative protein content. (**d**, **f**, **h**) PLB4 values are normalised to WT values. White bars, WT mice; black bars, PLB4 mice. Data show means + SEM. *p < 0.05, **p < 0.01

Evidence of failing glucose storage in mice expressing *hBACE1* was indicated by their reduced hepatic glycogen levels (Fig. 2a), increased hepatic triacylglycerol content (Fig. 2b) and inability to activate (i.e. dephosphorylate) GS in response to hyperglycaemic conditions (Fig. 2c,d). Heightened translational demand was also identified in PLB4 hepatocytes, suggested by increased phosphorylation of the rpS6 component of 40S ribosomes (Fig. 2c,d). Additionally, the ApoE, PTP1B and RBP4 lipometabolic regulators linked with obesity and insulin resistance [6, 24, 25] were upregulated in PLB4 mouse liver (Fig. 2c,d), suggesting decreased hepatic insulin sensitivity. Levels of these regulators were unchanged in white adipose tissue (WAT) and muscle (Fig. 2e–h).

### Dysregulated central and plasma lipid composition

The non-alcoholic fatty liver phenotype of PLB4 mice corresponded to that observed in human type 2 diabetes [26], and called for further investigation of putative changes in lipid metabolism. Altered lipid composition was recently proposed to predict ‘phenoconversion’ from mild cognitive impairment to Alzheimer’s disease, potentially offering a novel biomarker [27]. Hence, we applied a comparative global lipidomics approach and identified 321 altered species in PLB4 mice vs controls (see Table 1 for a summary and electronic supplementary material Tables 1–4 for details) comprising phospholipids, sphingomyelins and sphingolipids such as ceramides. Phospholipids such as phosphatidylethanolamine, lysophosphatidylethanolamine and phosphatidylserine were predominantly increased in both the brain and plasma; however only 11% of the altered lipid species were identical in both sample types. Fewer changes in lipid composition were detected in plasma samples than in brain samples.

**Table 1:** Summary of the brain and plasma lipid species altered in PLB4 mice

Lipid species	↑ Increased vs WT			↓ Decreased vs WT		
	Brain	Plasma	BvsP match, *n* (%)	Brain	Plasma	BvsP match, *n* (%)
Phospholipids	116	77	24 (12)	17	11	0
PC and lysoPC	40	31	14 (20)	7	6	0
PE and lysoPE	36	20	5 (9)	7	4	0
PG	6	1	0	0	0	0
PS	23	19	3 (7)	3	1	0
PI	10	5	2 (13)	0	0	NA
Other	1	1	0	0	0	NA
Sphingomyelin	10	10	7 (35)	1	0	0
Sphingolipids	29	8	0	3	6	0
Ceramides	20	7	0	3	3	0
Other	9	1	0	0	3	0
Diacylglycerols	10	3	2 (15)	1	1	0
Triacylglycerols	31	12	0	2	3	0
Total determined	196	110	33 (11)	24	21	0

Data show the numbers of lipids significantly (*p* < 0.05) up- or downregulated in 6-month-old PLB4 mice relative to age-matched WT controls

Brain vs plasma comparison (BvsP): overlap of altered lipid species between brain and plasma tissue in PLB4 mice; PC, phosphatidylcholine; PE, phosphatidylethanolamine; PG, phosphatidylglycerol; PI, phosphatidylinositol; PS, phosphatidylserine

### Reduced brain glucose metabolism

**Fig. 3:**
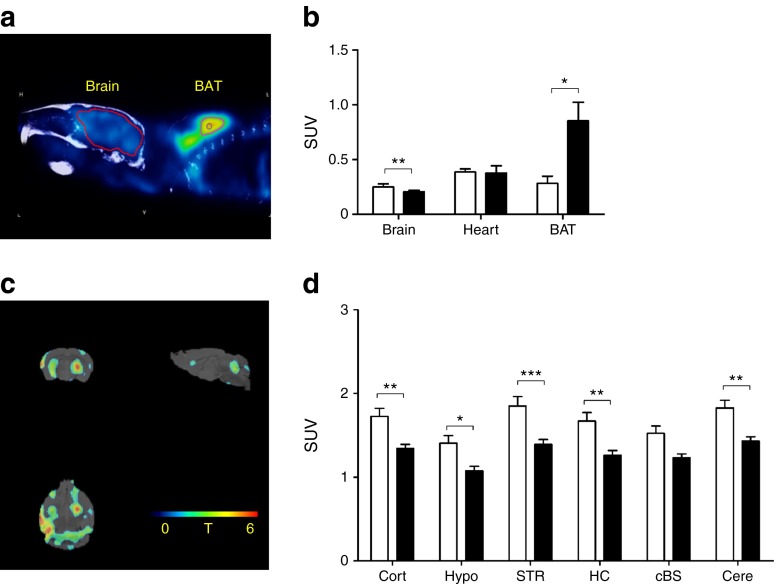
Abnormal glucose metabolism in PLB4 mice, as measured by ^18^FDG-PET imaging. (**a**) Representative scan illustrating glucose uptake based on ^18^FDG-PET imaging in brain and BAT. (**b**) Quantification of upper body glucose uptake for the brain, heart and BAT. (**c**) Statistical parametric mapping shows reduced regional *T* scores (0–6) for brain metabolism in PLB4 vs WT animals. (**d**) Quantification of regional brain glucose uptake in WT and PLB4 mice. cBS, caudal brainstem; Cort, cortex; Cere, cerebellum; HC, hippocampus; Hypo, hypothalamus; STR, striatum; SUV, standardised uptake value. White bars, WT mice; black bars, PLB4 mice. Data show means + SEM. *p < 0.5, **p < 0.01, ***p < 0.001

To further investigate whether the diabetic and metabolic phenotype of PLB4 mice was similar to that of human type 2 diabetes and dementia, we conducted an in vivo ^18^FDG-PET study (Fig. 3). and confirmed global cerebral hypometabolism in PLB4 mice vs WT controls at the symptomatic age of 6 months. In contrast to that of the brain, the metabolic activity of brown adipose tissue (BAT) was drastically enhanced, while cardiac metabolism did not differ between groups. As BAT activation is inversely related to body weight and obesity, the hypermetabolic readouts in PLB4 mice are consistent with their leaner phenotype [28] and indicative of a compensatory metabolic adjustment. Collectively, these data confirm that PLB4 mice have a diabetes-like phenotype.

### Abnormal neuronal insulin signalling

**Fig. 4:**
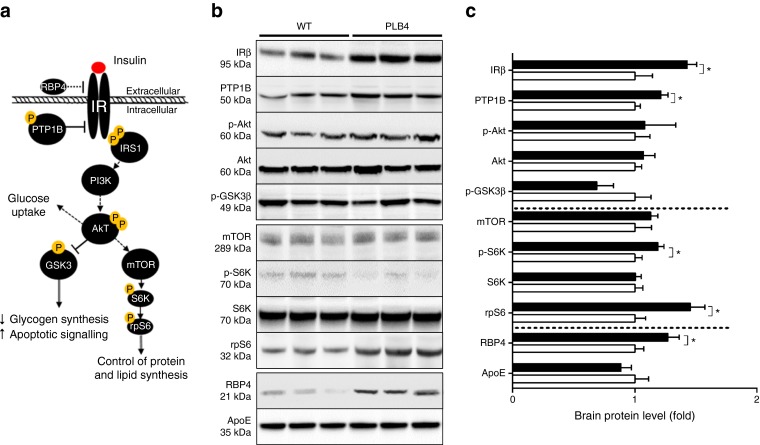
Defective neuronal insulin sensitivity in PLB4 mice. (**a**) Simplified schematic showing insulin signalling. (**b**) Representative immunoblots of insulin-related markers in brain lysates from 8-month-old PLB4 and WT mice and (**c**) quantification of their relative expression. White bars, WT mice; black bars, PLB4 mice. Data are means + SEM normalised to WT values. *p < 0.05

Since *hBACE1* was expression was forebrain specific, we next investigated whether neuronal insulin signalling was affected in symptomatic PLB4 mice by probing for markers of the insulin IR–Akt–GSK-3β transduction pathway (Fig. 4) in forebrain lysates of 8-month-old fasted mice. Total IR was upregulated in PLB4 vs WT mice, together with RBP4, thus corroborating links with insulin resistance [29]. PTP1B levels were also increased, indicating downregulation of central insulin and leptin signalling [6, 24]. There were no differences in brain levels of Akt between PLB4 and WT mice, but trends for decreased baseline GSK-3β phosphorylation in PLB4 mice indicated early neuronal disinhibition of this pro-apoptotic kinase. Targets downstream of mTOR were also altered: although levels of mTOR did not differ in PLB4 forebrain vs controls, we detected increased levels of rpS6 and its kinase S6K, implying an overall increase in translational demand consistent with elevated brain lipid synthesis in PLB4 mice.

### Altered hypothalamic melanocortin transcription and ER stress

**Fig. 5:**
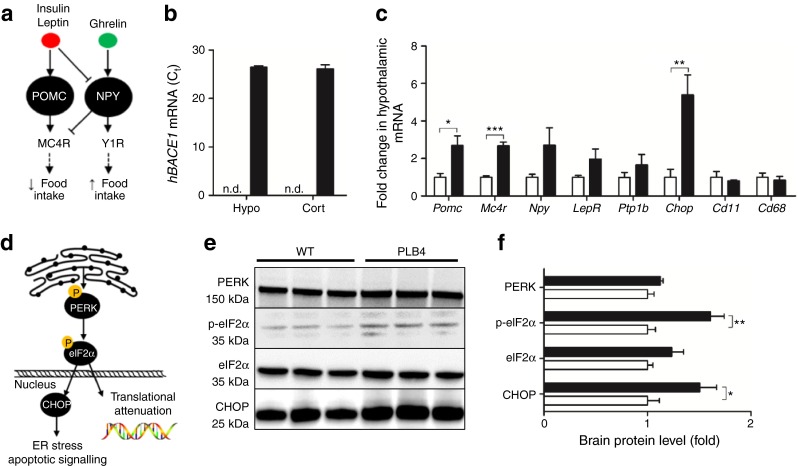
Hypothalamic pathology and neuronal ER stress induced by *hBACE1* knockin. (**a**) Simplified schematic showing anabolic and catabolic signalling in the hypothalamus. MC4R, melanocortin 4 receptor; NPY, neuropeptide Y; POMC, pro-opiomelanocortin; Y1R, neuropeptide Y1 receptor. (**b**) *BACE1* mRNA levels (raw C_t_ values) in PLB4 hypothalamic (Hypo) and cortical (Cort) brain fractions. Signal was not detected (n.d.) in WT mice. (**c**) Fold changes in the hypothalamic mRNA expression of genes related to appetite suppression (*Pomc*, *Mc4r*), appetite stimulation (*Npy*), leptin and insulin sensitivity (*LepR*, *Ptp1b*), ER stress (*Chop*), and microglial inflammation (*Cd11* and *Cd68*) in 8-month-old PLB4 mice. (**d**) Simplified schematic of the ER stress pathway. (**e**) Representative immunoblots of ER stress markers in brain lysates from 8-month-old mice and (**f**) quantification of their relative expression. White bars, WT mice; black bars, PLB4 mice. Data represent the means + SEM normalised to WT values. *p < 0.05, **p < 0.01, ***p < 0.001

As the hypothalamus is the regulatory brain centre for metabolic control, we reasoned that its malfunction was a possible cause of the failing insulin signalling and diabetic phenotype of PLB4 mice. Hypothalamic function was assessed via gene expression analysis of neuro- and polypeptides that regulate the hypothalamic–pituitary–adrenal axis (Fig. 5a–c). Transcription of the appetite-suppressing pro-opiomelanocortin (*Pomc*) gene was drastically increased in PLB4 mice compared with controls. POMC-positive neurons respond to leptin or insulin to reduce food intake and maintain energy homeostasis via secretion of alpha melanocyte-stimulating hormone (αMSH) and melanocortin receptor 4 (MC4R). A consistent increase in *Mc4r* gene expression was detected in the PLB4 vs WT hypothalamus, suggesting a shift toward anorectic signalling in mouse brains expressing *hBACE1*. Importantly, hypothalamic ER stress (see Fig. 5d) was confirmed by a drastic rise in *Chop* transcription in PLB4 tissue compared with controls. Corresponding protein levels were also increased (Fig. 5e,f), as was phosphorylated (i.e. activated) eIF2α, a major CHOP regulator. These changes were not associated with transcriptional alterations in inflammatory markers (microglial *Cd11* and *Cd68*).

## Discussion

Diabetic complications can lead to cognitive dysfunction and are acknowledged risk factors of Alzheimer’s disease, but little is known about the reverse scenario. Here, we demonstrate that the major amyloidogenic enzyme, BACE1, is sufficient to increase the risk of diabetes development when expressed in neurons only. Severe impairment in systemic glucose homeostasis and insulin sensitivity was evident in PLB4 mice from the age of 4 months onwards and progressively deteriorated with age. We previously reported that PLB4 mice develop mild cognitive deficits between 4 and 6 months. Our current findings therefore indicate that neuronal BACE1 induces global metabolic dysregulation along with brain inflammation and amyloidosis-related cognitive decline. Overall, the diabetic profile of PLB4 mice agrees with the improved glucose clearance and insulin sensitivity in mice lacking murine *Bace1* globally [17, 30], but pinpoints neuronal BACE1 as the major driver of this alteration.

Although both *Bace1* knockout and our *hBACE1* knockin mice were leaner compared with WT controls, there were differential effects on adiposity and leptin signalling. Deletion of *Bace1* decreased adiposity and improved sensitivity to leptin, while neuronal *hBACE1* expression promoted adipogenesis and induced hyperleptinaemia. Hence, it seems plausible that manipulation of (neuronal) BACE1 affects body weight via changing metabolic efficiency, adipose composition and signalling. Importantly, leptin production and signalling are regulated by neuronal PTP1B [24]. We propose that the elevated central PTP1B expression in PLB4 forebrains may explain the hyperleptinaemic profile and compensatory BAT hyperactivity in these mice, which confirms that leptin has opposing effects on WAT and BAT [31].

Further, while proinsulin synthesis appeared unchanged in PLB4 mice compared with controls, heightened levels of amylin indicate an attempt to downregulate hyperglycaemia in the insulin-deficient state, and suggests that pancreatic function was at least partially preserved in 8-month-old PLB4 mice. Glucose intolerance and the fatty liver phenotype in PLB4 mice were further associated with drastically elevated levels of serum GIP. Although GIP exhibits insulinotropic properties under physiological conditions, its incretin effects are thought to be blunted in the diabetic state, and elevated levels may promote fatty acid accumulation and induction of proinflammatory cytokines [32].

The fatty liver phenotype of PLB4 mice corresponded with high plasma triacylglycerol and elevated levels of phospholipids such as phosphatidylcholine and lysophosphatidylcholine, which are typically observed in type 2 diabetic patients [26]. Altered plasma lipid composition was also recently proposed to predict early memory impairments, thus potentially offering a novel approach to identify Alzheimer’s disease [27, 33, 34].

Although lipid accumulation was also affected in neuronal tissue from symptomatic PLB4 mice, there was a poor species match for plasma, suggesting that (1) plasma markers may not be indicative of changes in brain lipid composition and (2) that the increase in neuronal lipids probably originated from the CNS. PLB4 mice had pronounced elevation of several classes of phospholipids such as phosphatidylethanolamine, lysophosphatidylethanolamine and phosphatidylserine, which are major components of neuronal membrane bilayers. Such alterations are reported in human Alzheimer’s disease brains [35] and are proposed to affect mitochondrial function, signal transduction and receptor activation, hence interfering with neurotransmission and neuronal integrity [36, 37].

Importantly, brain ceramide levels were substantially increased in PLB4 mice compared with controls. This sphingomyelin precursor is of particular interest because it increases naturally with age [38] and at an early stage of human Alzheimer’s disease [39]. Furthermore, ceramides were previously shown to regulate BACE1 protein expression and promote APP β-site cleavage [40]. Although the mechanisms through which BACE1 may upregulate ceramide accumulation are largely unknown, initial evidence suggests that Aβ peptides may activate sphingomyelinase [41]. It therefore seems plausible that the introduction of BACE1 promotes ceramide biogenesis, and vice versa. In support of this, a recent study demonstrated that intracerebroventricular ceramide infusions induce lipotoxicity and hypothalamic ER stress associated with increased eIF2α and PERK phosphorylation, sympathetic inhibition, reduced weight gain and altered energy balance in rats [42].

Cerebral hypometabolism revealed via ^18^FDG-PET imaging is common in early dementia patients as well as diabetic patients with or without mild cognitive impairment [43], and may ultimately contribute to cognitive pathology. We found that reduced glucose utilisation in PLB4 mice was associated with poor neuronal insulin sensitivity. Altered IR and PTP1B expression occurred in PLB4 mice at an advanced stage of systemic insulin resistance, hepatic dysfunction and Aβ-associated cognitive impairment [13]. Similar elevations in neuronal PTP1B were observed in response to HFD feeding in other Alzheimer’s disease models [6], while deletion of neuronal PTP1B improved IR signalling and protected against HFD-induced obesity and insulin resistance [24].

Increased S6K phosphorylation in PLB4 vs WT brains and elevated expression of its substrate, rpS6, suggest an increased demand for protein and lipid synthesis. Additionally, the ribosomal element is regulated by eIF2, offering an alternative route for its modification [44]. Elevated rpS6 phosphorylation along with increased brain RBP4 levels were previously found in Alzheimer’s disease mice on a HFD [6]. Neuronal pathways that mediate RBP4’s action and toxicity are yet to be investigated, but have been linked to proinflammatory cytokines in macrophages and to activation of JNK, a major ER stress kinase [29].

Recent studies revealed reduced hypothalamic volume and accelerated atrophy of orexin neurons in early Alzheimer’s disease [45, 46]. In contrast to diet-induced obese and diabetic models, PLB4 mice displayed increased *Pomc* and *Mc4r* mRNA levels, suggesting a hypothalamic shift toward appetite suppression and increased energy expenditure. The increase in hypothalamic *Mc4r* and *Pomc* transcription in PLB4 mice further contrasts with the recently demonstrated Aβ-oligomer-induced elevation in *Npy* mRNA (but not in *Pomc* mRNA), which was not associated with changes in circulating leptin levels [47]. Here, elevated melanocortin transcription in the hypothalamus of PLB4 mice may be a downstream effect of persistently increased circulating levels of leptin and systemic hyperglycaemia.

An advanced state of ER stress was confirmed in the hypothalamus of PLB4 mice, resembling that induced by HFD feeding [48]. Pharmacologically induced hypothalamic ER stress resulted in systemic diabetes in mice [49]; this was also recently illustrated for Aβ oligomer infusions [47]. The pathology of PLB4 mice therefore agrees with a scenario of BACE1-driven elevations of the ER stress marker CHOP and the protein translation regulator eIF2α, which suggest induction of an integrated stress response. Mechanistically, neuronal expression of human BACE1 may promote hypothalamic ER stress via ceramide lipotoxicity, activation of eIF2α and an integrated stress response [42], in addition to driving Aβ production [13].

In conclusion, we demonstrate that neuronal expression of human BACE1 causes systemic diabetic complications. We propose that increased levels of central BACE1 promotes metabolic disturbance via inducing hypothalamic impairment, ER stress, and Aβ and lipid accumulation, leading to neuronal damage, insulin resistance, hepatic deficits and global glucose dyshomeostasis. The comorbid phenotype of the PLB4 mouse provides insight into the complex mechanistic interactions between diabetes and Alzheimer’s disease. As an extension to the hypothesis that diabetic complications promote the onset and progression of Alzheimer’s disease, we suggest that the reverse scenario may also apply.

## Electronic supplementary material

Below is the link to the electronic supplementary material.

ESM Table 1 (PDF 363 kb)

ESM Table 2 (PDF 126 kb)

ESM Table 3 (PDF 274 kb)

ESM Table 4 (PDF 124 kb)

## Abbreviations

Akt: Protein kinase B
ApoE: Apolipoprotein ε
APP: Amyloid precursor protein
BACE1: β-Secretase 1
BAT: Brown adipose tissue
CHOP: C/EBP homologous protein
CNS: Central nervous system
CT: Computed tomography
eIF2α: Eukaryotic initiation factor 2α
ER: Endoplasmic reticulum
^18^FDG-PET: 2-[^18^F]Fluoro-2-deoxy-d-glucose positron emission tomography
GIP: Glucose-dependent insulinotropic peptide
GSIS: Glucose-stimulated insulin secretion
GTT: Glucose tolerance test
*hBACE1*: Human *BACE1* gene
HFD: High-fat diet
IRβ: Insulin receptor β
mTOR: Mammalian target of rapamycin
GS: Glycogen synthase
GSK3β: Glycogen synthase kinase-3 β
rpS6: Ribosomal protein S6
S6K: p70-S6 kinase
PERK: Protein kinase RNA-like ER kinase
PTP1B: Protein tyrosine phosphatase 1B
RBP4: Retinol-binding protein 4
STZ: Streptozotocin
WAT: White adipose tissue
WT: Wild-type
