# Supplementary material for: Neuronal human BACE1 knockin induces systemic diabetes in mice
Source: Diabetologia. 2016 May 2;59:1513–23. doi: 10.1007/s00125-016-3960-1 (PMC4901117; doi:10.1007/s00125-016-3960-1)
Supplement: Supplementary file 1 — (PDF 363 kb) [file 125_2016_3960_MOESM1_ESM.pdf]

ESM Table 1

Identification	Ionisation Mode	Adducts	m/z	Mass Error (ppm)	Anova (p)	Fold Change
**Phospholipids**						
**PC and LysoPC**						
PC(16:0)	+	M+H, M+Na	496.3400	0.68	0.009	1.21
PC(18:1)	+	M+H, M+Na	522.3557	1.328	0.008	1.21
PC(22:6)	+	M+H	568.3400	0.41	0.002	1.33
PC(32:0)	-	M-H, M+FA-H	732.5550	0.22	0.020	1.24
PC(34:0)	-	M+FA-H	806.5934	2.27	0.004	1.12
PC(34:1)	+	M+H, M+Na, M+K	760.5848	-0.39	0.005	1.13
PC(34:2)	+	M+Na, M+K	780.5519	0.97	0.023	1.18
PC(34:3)	+	M+H	756.5542	0.47	0.022	1.23
PC(36:1)	+	M+Na, M+K, M+H	788.6163	-0.86	0.008	1.16
PC(36:3)	+	M+H	784.5852	0.18	0.0004	1.35
PC(36:4)	+	M+H, M+K	782.5694	0.02	0.0007	1.27
	-	M+FA-H, M-H , M+Na-2H, M-H2O-H	778.5776	-0.64	0.010	1.17
PC(36:5)	+	M+H, M+Na	780.5543	0.56	0.0002	1.31
PC(38:3)	+	M+H	812.6162	-0.29	0.0008	1.33
PC(38:4)	+	M+H	810.5996	-1.39	0.0009	1.26
PC(38:5)	-	M+FA-H, M-H2O-H	788.5291	2.06	0.002	1.14
PC(38:6)	+	M+H, M+Na, M+K	806.5690	-0.64	0.0014	1.26
	-	M+FA-H, M-H2O-H,	786.5099	2.48	0.001	1.35
PC(38:7)	+	M+H, M+Na	804.5540	0.20	0.005	1.29
PC(38:8)	+	M+H	802.5374	-0.98	0.001	1.71
PC(40:4)	+	M+H, M+Na, M+K	838.6304	-2.06	0.001	1.24
PC(40:6)	+	M+H, M+Na, M+K	834.5998	-1.15	0.002	1.21
PC(40:7)	+	M+H, M+Na, M+K	832.5842	-0.89	0.0007	1.24
PC(40:8)	-	M+FA-H	874.5626	2.71	0.0007	1.43
PC(42:10)	+	M+Na, M+K, M+H	854.5699	0.54	0.0001	1.40
	-	M+FA-H	898.5622	2.10	4.64E-05	1.36
PC(42:4)	+	M+Na, M+K, M+H	866.6628	0.41	0.038	1.26
PC(42:5)	+	M+Na	886.6284	-1.41	0.007	1.23
	-	M-H	862.6315	-1.93	0.037	1.41
PC(42:6)	+	M+H	862.6345	3.38	0.0009	1.28
PC(42:7)	+	M+Na	882.5981	-0.22	7.85E-05	1.33
PC(42:8)	+	M+Na	880.5853	3.07	0.0006	1.50
PC(42:9)	-	M+FA-H	900.5778	2.05	1.52E-05	1.97
PC(44:12)	+	M+H, M+Na, M+K	878.5697	0.35	0.0009	1.29
	-	M+FA-H	922.5562	-4.74	0.001	1.22
PC(52:4)	+	M+Na	1028.8012	-0.54	0.016	1.25
PC(O-32:0)	+	M+H, M+Na	720.5904	0.261	0.0007	1.29

	-	M+FA-H	764.5833	3.01	0.039	1.14
PC(O-34:0)	+	M+H, M+Na	748.6215	1.29	0.013	1.24
PC(O-34:1)	+	M+H	746.6065	0.89	0.0027	1.28
PC(O-42:2)	+	M+H	856.7155	0.14	0.006	1.56
PC(P-32:0)	+	M+Na	740.5574	1.32	0.0002	1.16
PC(P-34:4)	+	M+H	738.5449	2.25	0.009	1.26
PC(P-36:1)	+	M+H	772.6224	1.09	0.031	1.20
PC(P-38:5)	+	M+H, M+Na	792.5897	-0.70	0.0002	1.35
PC(P-40:6)	+	M+H	818.6072	1.62	0.002	1.292
**PE and LysoPE**						
PE(16:0)	-	M-H	452.2789	1.44	0.004	1.20
PE(18:0)	+	M+H	482.3243	0.27	0.014	1.23
	-	M-H	480.3101	1.06	0.004	1.23
PE(18:1)	-	M-H	478.2944	0.99	0.042	1.24
PE(20:1)	-	M-H	506.3256	0.84	0.007	1.19
PE(20:4)	+	M+H	502.2932	0.74	0.037	1.22
PE(22:4)	+	M+H	530.3252	1.95	0.003	1.35
	-	M-H	528.3101	1.08	0.048	1.79
PE(22:6)	+	M+H	526.2929	0.05	0.006	1.39
PE(34:0)	+	M+H, M+Na	734.5696	-1.06	0.001	1.19
	-	M-H	718.5408	2.17	0.000	1.18
PE(36:1)	-	M-H	744.5567	2.43	0.003	1.12
PE(36:3)	+	M+H, M+Na	742.5386	0.62	0.017	1.32
PE(36:4)	+	M+Na, M+K, M+H	740.5236	-0.57	0.010	1.23
	-	M-H	738.5100	2.80	0.015	1.11
PE(36:5)	+	M+H	738.5078	1.20	0.000	1.59
PE(38:0)	-	M-H	774.6009	-1.22	0.040	1.28
PE(38:3)	-	M-H	768.5574	3.26	0.000	1.29
PE(38:5)	-	M-H	764.5250	1.82	0.001	1.62
PE(38:6)	+	M+H, M+Na	764.5233	0.96	0.001	1.30
PE(40:0)	+	M+Na	826.6279	-2.11	0.009	1.97
PE(40:10)	+	M+Na	810.5020	-3.12	0.022	1.65
PE(40:4)	-	M-H	794.5724	2.30	0.000	1.18
PE(40:5)	+	M+Na	816.5498	-1.97	0.003	2.42
PE(40:6)	+	M+H, M+K, M+Na	792.5545	0.84	0.003	1.24
	-	M-H	790.5415	2.83	0.003	1.14
PE(42:5)	-	M-H	820.5877	1.91	0.007	1.25
PE(42:6)	+	M+H, M+Na	820.5842	-1.16	0.007	1.35
PE(42:7)	+	M+H	818.5688	-0.87	0.028	1.37
	-	M-H	816.5573	2.98	0.002	1.17
PE(42:8)	-	M-H	814.5410	2.12	0.000	1.83
PE(42:9)	-	M-H	812.5256	2.47	0.000	1.48

PE(44:12)	+	M+H	836.5242	2.04	0.001	1.28
PE(O-36:0)	-	M-H	732.5930	2.36	0.008	1.32
PE(P-16:0)	-	M-H	436.2838	1.01	0.005	1.27
PE(P-18:0)	+	M+H	466.3293	0.20	0.005	1.30
PE(P-36:3)	-	M-H	724.5319	4.40	0.007	1.20
PE(P-36:4)	+	M+H	724.5289	1.79	0.001	1.30
PE(P-38:4)	+	M+H	752.5603	1.79	0.004	1.27
	-	M-H	750.5461	2.31	0.006	1.15
PE(P-38:6)	+	M+H, M+Na	748.5293	2.20	0.001	1.30
PE(P-40:4)	+	M+H, M+Na	780.5911	1.12	0.011	1.28
PE(P-40:6)	+	M+H, M+Na, M+K	776.5606	2.16	0.002	1.27
**PG**						
PG(38:4)	+	M+H	799.5507	2.86	0.033	1.41
	-	M-H	797.5358	2.45	0.007	1.26
PG(38:5)	-	M-H	795.5198	2.10	0.014	1.67
PG(40:6)	-	M-H	821.5334	-0.46	0.043	1.39
PG(42:10)	-	M-H	841.5040	1.73	0.006	1.27
PG(42:9)	-	M-H	843.5196	1.69	0.001	1.94
PG(44:12)	-	M-H	865.5040	1.76	0.006	1.19
**PS**						
PS(36:2)	+	M+H	788.5424	-1.64	0.006	1.24
	-	M-H	786.5310	2.51	0.022	1.12
PS(36:3)	+	M+H	786.5314	4.32	0.036	1.34
	-	M-H	784.5146	1.49	0.012	1.21
PS(37:0)	-	M-H	804.5777	2.10	0.012	1.10
PS(37:2)	-	M-H	800.5461	1.70	0.006	1.61
PS(38:0)	-	M-H	818.5940	2.88	0.034	1.14
PS(38:3)	+	M+H	814.5593	0.04	0.005	1.22
	-	M-H	812.5443	-0.44	0.011	1.15
PS(38:4)	+	M+H, M+Na	812.5448	1.40	0.035	1.16
PS(39:0)	-	M-H	832.6094	2.47	0.006	1.13
PS(39:1)	-	M-H, M+Na-2H	830.5934	2.13	0.017	1.12
PS(39:4)	-	M-H	824.5467	2.45	0.011	1.18
PS(40:6)	+	M+H, M+Na	836.5432	-0.56	0.001	1.29
PS(40:7)	+	M+H	834.5290	1.22	0.007	1.19
PS(41:2)	-	M-H	856.6092	2.25	0.0002	1.23
PS(41:5)	+	M+Na	874.5546	-2.60	0.006	1.72
PS(41:6)	+	M+Na, M+K	872.5389	-2.72	0.021	1.19
	-	M-H	848.5466	2.19	0.004	1.17
PS(42:10)	-	M-H	854.4996	2.11	0.001	1.43
PS(42:5)	-	M-H	864.5790	3.44	0.014	1.24

PS(42:8)	+	M+Na	882.5289	3.86	0.001	1.64
PS(43:6)	-	M-H	876.5774	1.60	0.001	1.16
PS(44:10)	+	M+H, M+Na	884.5468	3.61	0.001	1.35
PS(44:12)	-	M-H	878.4971	-0.75	0.003	1.52
PS(49:2)	-	M-H	828.5774	1.62	0.0003	1.26
PS(P-40:0)	-	M-H	830.6313	3.93	0.030	1.19
**PI**						
PI(36:3)	-	M-H	859.5327	-1.77	0.007	1.43
PI(36:4)	-	M-H	857.5201	1.77	0.000	1.21
PI(37:4)	-	M-H	871.5360	2.08	0.024	1.18
PI(38:5)	-	M-H	883.5358	1.82	2.61E-04	1.20
PI(38:6)	-	M-H	881.5202	1.90	0.026	1.12
PI(40:4)	-	M-H	913.5832	2.23	0.016	1.16
PI(40:5)	-	M-H	911.5673	1.96	0.006	1.18
PI(40:8)	-	M-H	905.5206	2.30	5.87E-06	1.90
PI(42:10)	-	M-H	929.5213	2.91	0.001	2.06
PI(P-38:4)	-	M-H	869.5566	1.88	4.67E-05	1.42
**Other PL**						
CL(68:2)	+	M+NH4	1423.0414	2.88	0.047	1.69
**Sphingomyelin**						
SM(d32:1)	+	M+H	675.5442	0.88	0.033	1.21
SM(d34:1)	+	M+H, M+Na, M+K	703.5757	1.09	0.003	1.24
	-	M+FA-H	747.5674	2.35	0.001	1.16
SM(d34:2)	+	M+H	701.5560	-4.60	0.001	1.30
SM(d35:1)	+	M+H, M+Na	717.5873	-4.51	0.001	1.31
	-	M-H	715.5780	2.81	0.001	1.16
SM(d36:0)	+	M+H	733.6222	0.47	0.005	1.24
SM(d36:1)	+	M+H, M+Na, M+K	731.6072	1.39	0.002	1.18
	-	M+FA-H	775.5960	-1.55	0.005	1.14
SM(d36:2)	+	M+H, M+K, M+Na	729.5913	1.02	0.000	1.45
	-	M+FA-H	773.5818	0.44	3.68E-04	1.32
SM(d38:1)	+	M+H, M+Na	759.6387	1.55	0.009	1.20
SM(d40:2)	+	M+H	785.6528	-0.42	0.019	1.24
	-	M+FA-H	829.6469	3.58	0.020	1.21
SM(d42:3)	-	M+FA-H	855.6625	3.46	0.023	1.18
**Sphingolipids**						
Cer(d36:0)	-	M-H	566.5528	1.81	0.039	1.19
Cer(d36:2)	-	M-H, M+FA-H	562.5214	1.58	0.011	1.23
Cer(d37:1)	-	M-H	578.5530	2.06	0.037	1.30

Cer(d38:0(2OH)	-	M-H	610.5790	1.63	0.001	1.45
Cer(d40:0(2OH))	-	M-H	638.6102	1.45	4.96E-04	1.30
Cer(d40:0)	+	M+H	624.6295	0.78	0.045	1.23
Cer(d40:1)	+	M+Na	644.5982	4.73	0.009	1.51
HexCer(d34:1)	-	M-H, M+FA-H	698.5592	2.25	0.001	1.35
HexCer(d35:1)	-	M-H	712.5755	3.04	0.033	1.74
HexCer(d36:0)	+	M+H	730.6200	1.09	0.007	1.40
HexCer(d36:0)	-	M-H	728.6041	-0.65	0.007	1.17
HexCer(d36:1)	-	M-H, M+Na-2H	748.5303	2.33	0.021	1.10
HexCer(d36:2(2OH))	-	M-H	740.5698	2.10	0.004	1.40
HexCer(d37:1)	-	M-H	740.6066	2.70	0.033	1.25
HexCer(d38:0)	-	M-H	756.6329	-3.92	0.009	1.23
HexCer(d38:1)	-	M-H, M+FA-H	754.6203	0.10	0.049	1.12
HexCer(d40:1)	-	M-H	782.6504	-1.40	0.024	1.27
HexCer(d40:2(2OH))	-	M-H, M+FA-H	796.6310	0.34	9.75E-05	1.23
HexCer(d40:2)	-	M-H, M+FA-H	780.6377	2.37	0.009	1.24
HexCer(d41:2)	-	M-H, M+FA-H	794.6539	2.98	0.027	1.19
C16 Sulfatide	-	M-H	778.5159	1.91	0.003	1.24
C22 Sulfatide	-	M-H	862.6092	1.01	0.002	1.27
NeuAcα2-3Galβ1-4Glcβ-Cer(d36:1)	-	M-H	1179.7387	1.28	0.009	1.16
NeuAcα2-6GalNAcβ1-4Galβ1-4Glcβ-Cer(d36:1)	-	M-H	1382.8166	0.06	0.005	1.24
NeuAcα2-8NeuAcα2-3Galβ1-4Glcβ-Cer(d36:1)	-	M-H	1470.8332	0.38	0.037	1.24
GalNAcβ1-3Galα1-3Galα1-3Galα1-3Galα1-4Galβ1-4Glcβ-Cer(d44:1)	-	M-H	1852.0629	2.84	0.008	1.06
Galα1-3(Fucα1-2)Galβ1-3GalNAcβ1-3Galα1-3Galβ1-4Glcβ-Cer(d42:1)	-	M-H	1808.0286	-1.54	0.005	1.10
Galα1-3(Fucα1-2)Galβ1-4(Fucα1-3)GlcNAcβ1-3Galβ1-4Glcβ-Cer(d42:2)	-	M-H, M+Na-2H	1811.9962	-3.68	0.002	1.39
Galβ1-3GalNAcβ1-4(KDNα2-3)Galβ1-4Glcβ-Cer(d44:1)	-	M-H	1615.9616	-3.95	0.041	13.62
**Diacylglycerols**						
DG(18:4)	+	M+H	641.5119	-3.20	0.004	1.31
DG(35:0)	+	M+K	649.5195	4.54	0.001	1.37
DG(35:1)	+	M+K	647.5011	-0.03	0.009	2.14
DG(36:4)	+	M+Na, M+K	655.4703	0.88	0.016	1.41
DG(38:4)	+	M+NH4, M+K, M+Na, M+H	667.5276	0.61	0.011	1.20
DG(38:5)	+	M+Na, M+K	681.4859	0.28	0.013	1.39

DG(40:4)	+	M+Na, M+K	695.5521	1.26	0.043	1.30
DG(40:6)	+	M+NH4, M+Na, M+K	691.5282	0.55	0.009	1.24
DG(40:7)	+	M+Na, M+K	705.4863	2.02	0.035	1.36
DG(42:10)	+	M+H, M+K	689.5137	-0.38	0.025	1.67
**Triacylglycerols**						
TG(43:2)	+	M+Na	755.6135	-3.47	0.008	1.27
TG(47:6)	-	M-H	779.6219	3.09	0.004	1.26
TG(50:1)	+	M+Na, M+K, M+NH4	871.7157	0.69	0.014	1.16
TG(51:4)	-	M+FA-H	885.7149	-4.82	0.001	1.48
TG(52:4)	+	M+Na, M+K, M+NH4	893.7016	2.47	0.006	1.28
TG(52:5)	+	M+K	891.6867	3.35	0.003	1.37
TG(52:6)	+	M+Na, M+K	889.6682	0.06	0.004	1.25
TG(54:4)	+	M+Na, M+K	921.7313	2.66	0.002	1.24
TG(54:5)	+	M+NH4, M+K, M+Na	919.7162	1.22	0.007	1.20
TG(54:6)	+	M+Na, M+K, M+NH4	917.7002	0.82	4.71E-04	1.28
TG(54:7)	+	M+Na, M+K	915.6844	0.68	0.001	1.28
TG(56:6)	+	M+NH4, M+K, M+Na	945.7319	1.29	0.001	1.30
TG(56:7)	+	M+NH4, M+K, M+Na	943.7157	-2.45	2.84E-04	1.33
TG(56:8)	+	M+NH4, M+K, M+Na	941.7002	0.86	0.001	1.33
TG(56:9)	+	M+K	939.6871	3.61	0.004	1.80
TG(57:6)	+	M+NH4	938.8170	-0.07	0.003	1.43
TG(58:10)	+	M+Na, M+K	965.7018	2.56	2.62E-04	1.38
TG(58:12)	+	M+Na, M+K	945.6939	-0.42	0.004	1.32
TG(58:6)	+	M+NH4	952.8304	-2.51	0.041	1.74
TG(58:7)	+	M+NH4, M+K, M+Na	971.7478	1.54	0.004	1.24
TG(58:9)	+	M+K	967.7157	0.61	4.30E-04	1.44
TG(59:6)	+	M+NH4	966.8479	-0.52	0.004	1.50
TG(59:7)	+	M+NH4	964.8281	-4.93	0.010	1.33
TG(60:10)	+	M+Na, M+K	993.7308	0.01	4.42E-04	1.37
TG(60:11)	+	M+Na, M+K, M+NH4	991.7226	1.28	1.61E-04	1.31
TG(60:12)	+	M+Na, M+K, M+NH4	989.7002	1.04	2.32E-04	1.38
TG(60:8)	+	M+Na, M+K	997.7637	1.66	0.009	1.47
TG(62:12)	+	M+Na, M+K, M+NH4	1017.7321	1.32	0.002	1.33
TG(62:13)	+	M+NH4, M+K, M+Na	1015.7136	-1.23	4.47E-04	1.44
TG(62:14)	+	M+H	975.7435	-0.18	0.026	2.57
TG(66:18))	+	M+K	1061.7025	2.95	0.001	1.88

Table ESM 1. Lipid species increased in the brain tissue from *hBace1* knock-in mice compared to WT controls. Shown are specific lipid species, ionisation mode (+ positive or - negative), adducts, m/z values (mass/mass spectrum charge number), mass error, p values obtained from ANOVA analysis and fold-change compared to WT controls. Abbreviations: *PC* phosphatidylcholine (>50% of total phospholipids), *PE* phosphatidylethanolamine, *PI* phosphatidylinositol, *PL* phospholipids and *PS* phosphatidylserine.
