# Supplementary material for: Neuronal human BACE1 knockin induces systemic diabetes in mice
Source: Diabetologia. 2016 May 2;59:1513–23. doi: 10.1007/s00125-016-3960-1 (PMC4901117; doi:10.1007/s00125-016-3960-1)
Supplement: Supplementary file 2 — (PDF 126 kb) [file 125_2016_3960_MOESM2_ESM.pdf]

ESM Table 2

Identification	Ionisation Mode	Adducts	m/z	Mass Error (ppm)	Anova (p)	Fold Change
**Phospholipids**						
**PC and LysoPC**						
PC(24:0)	+	M+H	608.4653	0.54	0.014	1.21
PC(30:1)	+	M+H	704.5233	1.12	2.30E-08	1.62
PC(32:2)	+	M+H	730.5385	0.47	0.001	1.16
PC(34:4)	+	M+H	754.5375	-0.90	0.001	1.29
PC(44:1)	+	M+Na	922.7261	2.90	0.025	1.42
PC(44:2)	+	M+Na	920.7121	4.75	0.035	1.31
PC(O-30:0)	+	M+H, M+Na	692.5588	-0.63	5.88E-06	1.22
**PE and LysoPE**						
PE(32:0)	+	M+H	692.5233	1.17	3.43E-05	1.80
	-	M-H	690.5095	2.33	9.13E-05	1.32
PE(32:1)	+	M+H	690.5089	2.91	0.001	1.14
PE(34:1)	+	M+H	718.5386	0.63	0.001	1.22
PE(42:1)	+	M+H	830.6593	-4.95	0.013	1.17
PE(O-40:0)	+	M+H	812.6519	-1.08	0.024	1.16
PE(P-32:1)	+	M+H	674.5134	2.14	2.79E-04	1.27
PE(P-42:1)	+	M+H	814.6690	0.67	0.026	1.26
**PS**						
PS(41:1)	+	M+H, M+Na	860.6386	4.80	0.014	1.21
PS(42:4)	+	M+Na	890.5845	-4.27	0.029	1.14
PS(P-30:1)	+	M+NH4	707.4938	-4.70	0.008	1.25
**Sphingomyelin**						
SM(d42:1)	+	M+H, M+Na	815.7000	-1.55	0.009	1.23
**Sphingolipids**						
Cer(d32:1)	+	M+H	650.6443	-0.52	0.029	1.68
Cer(d42:0)	+	M+H	652.6599	-0.53	0.012	1.19
HexCer(d44:2)	+	M+H	838.7122	-1.06	0.018	2.11
**Diacylglycerols**						
DG(32:0)	+	M+H	569.5152	2.19	0.037	1.32
**Triacylglycerols**						
TG(45:5)	+	M+NH4	772.6418	-4.26	0.045	1.88
TG(56:1)	+	M+NH4	934.8803	0.64	0.020	1.15

Table ESM 2. Lipid species decreased in the brain tissue from hBace1 knock-in mice compared to WT controls. Shown are specific lipid species, ionisation mode (+ positive or - negative), adducts, m/z values

(mass/mass spectrum charge number), mass error, p values obtained from ANOVA analysis and fold-change compared to WT controls. Abbreviations: *PC* phosphatidylcholine (>50% of total phospholipids), *PE* phosphatidylethanolamine, *PI* phosphatidylinositol, *PL* phospholipids and *PS* phosphatidylserine.
