# Supplementary material for: Neuronal human BACE1 knockin induces systemic diabetes in mice
Source: Diabetologia. 2016 May 2;59:1513–23. doi: 10.1007/s00125-016-3960-1 (PMC4901117; doi:10.1007/s00125-016-3960-1)
Supplement: Supplementary file 3 — (PDF 274 kb) [file 125_2016_3960_MOESM3_ESM.pdf]

ESM Table 3

Identification	Ionisation Mode	Adducts	m/z	Mass Error (ppm)	Anova (p)	Fold Change
**Phospholipids**						
**PC and LysoPC**						
PC(18:0)	+	M+H, M+Na, M+K	524.3714	0.68	0.011	1.24
PC(20:5)	+	M+H, M+Na	542.3249	1.48	0.018	2.25
	-	M+FA-H	586.3171	3.83	0.015	2.01
PC(22:4)	+	M+H	572.3719	1.46	0.014	1.33
	-	M+FA-H	616.3640	3.60	0.001	2.04
PC(22:5)	+	M+H	570.3564	1.78	0.024	1.51
	-	M+FA-H	614.3487	4.05	0.041	1.34
PC(24:0)	+	M+H	608.4657	1.17	0.049	1.40
	-	M+FA-H	652.4586	4.39	0.014	1.40
PC(36:1)	-	M-H, M+FA-H	832.6107	4.27	0.019	1.41
PC(36:2)	+	M+H, M+K	786.6008	0.02	0.022	1.52
	-	M+FA-H	830.5949	4.08	0.010	1.30
PC(36:4)	+	M+H, M+Na, M+K	782.5693	-0.20	0.033	1.18
PC(36:5)	+	M+Na, M+K, M+NH4	802.5367	1.29	0.004	1.56
PC(36:6)	+	M+NH4, M+K, M+H	778.5391	4.33	0.041	1.28
PC(38:4)	+	M+H	810.6018	1.32	0.032	1.38
PC(38:5)	+	M+H	808.5838	-1.57	0.003	1.27
PC(38:6)	+	M+H	806.5695	0.03	0.024	1.63
PC(38:7)	+	M+Na, M+K	826.5374	2.06	0.012	1.36
	-	M+FA-H	848.5483	4.46	0.024	1.20
PC(40:3)	+	M+Na	862.6292	-0.55	0.008	6.59
PC(40:5)	+	M+NH4	853.6441	1.40	0.001	1.50
PC(40:6)	+	M+H, M+Na, M+K	834.5996	0.10	0.007	1.27
	-	M+FA-H	878.5942	3.02	0.011	1.41
PC(40:7)	+	M+Na, M+K	854.5675	-3.23	0.007	1.34
PC(40:8)	+	M+Na, M+K, M+H	830.5695	1.52	7.54E-06	1.60
	-	M+FA-H	874.5640	4.33	2.16E-06	1.56
PC(40:9)	-	M+FA-H	872.5485	4.60	0.008	6.75
PC(42:10)	+	M+H, M+K	854.5698	0.38	0.000	1.90
PC(42:5)	-	M+Cl	898.6105	0.85	0.031	1.19
PC(42:7)	+	M+H, M+Na	860.6150	-1.61	0.049	1.17
PC(42:8)	+	M+H, M+Na	858.5975	-3.81	0.001	1.36
PC(44:12)	+	M+H, M+Na	878.5705	1.23	3.38E-04	1.80
PC(O-16:0)	+	M+H, M+Na	482.3612	1.52	0.008	1.20
	-	M+FA-H	526.3532	3.68	0.029	1.40
PC(O-36:4)	+	M+H	768.5871	-4.05	0.011	1.19
	-	M+FA-H	812.5846	4.56	0.028	1.25
PC(P-18:1)	+	M+H	506.3620	3.00	0.005	1.36

PC(P-32:1)	+	M+H	716.5588	-0.09	0.037	1.45
PC(P-34:0)	+	M+H	746.6061	0.34	0.005	1.24
PC(P-38:4)	-	M+FA-H	838.5973	0.72	0.016	1.26
**PE and LysoPE**						
PE(22:1)	+	M+H	536.3717	1.23	0.005	1.34
PE(22:5)	-	M-H	526.2956	3.17	0.017	2.05
PE(36:3)	+	M+Na	764.5209	1.07	0.004	1.61
PE(38:1)	+	M+H	774.6017	1.24	0.005	1.17
PE(38:2)	-	M-H	770.5736	4.01	0.007	1.32
PE(38:5)	+	M+H	766.5375	-0.86	0.009	1.52
PE(40:1)	+	M+H	802.6323	0.36	0.002	1.29
PE(40:2)	+	M+H, M+Na	800.6172	3.85	0.005	1.35
PE(40:4)	+	M+Na	818.5663	-0.91	0.005	1.33
PE(40:5)	+	M+Na	816.5508	-0.77	0.019	1.23
PE(40:8)	-	M-H	786.5117	4.73	0.002	2.25
PE(42:3)	+	M+K	864.5876	-0.35	0.012	1.27
PE(44:10)	-	M-H	838.5431	4.66	0.001	2.94
PE(44:2)	+	M+H	856.6799	1.05	0.043	1.32
PE(O-18:0)	-	M-H	466.3317	3.01	0.026	1.39
PE(P-20:0)	-	M-H	492.3477	3.46	0.031	1.52
PE(P-34:1)	+	M+NH4	719.5713	2.26	7.50E-05	1.65
PE(P-36:0)	-	M-H	730.5748	-1.10	0.007	1.59
PE(P-38:3)	+	M+NH4, M+Na	776.6106	4.58	0.041	1.21
PE(P-40:4)	-	M-H, M+FA-H	824.5485	-3.98	0.004	1.37
**PG**						
PG(42:3)	-	M-H	855.6145	2.82	0.028	1.41
**PS**						
PS(22:0)	-	M-H	580.3639	3.32	0.003	2.16
PS(34:0)	+	M+NH4	781.5668	-4.37	0.037	1.20
PS(35:1)	+	M+K	786.4678	-0.52	0.024	1.20
PS(36:1)	+	M+NH4	807.5847	-1.41	0.002	1.38
PS(37:3)	-	M-H	798.5323	4.12	0.005	1.73
PS(39:1)	-	M-H	830.5946	3.56	0.024	1.72
PS(40:4)	-	M-H	838.5642	4.57	0.008	1.16
PS(41:3)	+	M+Na, M+K	878.5861	-3.20	0.021	1.46
PS(41:4)	+	M+Na	876.5699	-3.05	0.007	1.13
PS(41:6)	+	M+Na	872.5390	-2.60	0.015	1.28
PS(41:7)	+	M+Na	870.5249	-0.77	0.004	1.60
PS(42:4)	+	M+Na	890.5876	-0.67	0.003	1.46
	-	M-H	866.5937	2.40	3.80E-04	2.11

PS(43:6)	-	M-H	876.5781	2.36	0.007	1.44
PS(44:7)	+	M+H	890.5936	3.45	0.016	1.61
PS(O-38:0)	+	M+H	512.3353	1.22	0.028	5.28
PS(P-36:0)	+	M+H	776.5823	2.96	4.07E-06	2.26
PS(P-36:1)	+	M+H	774.5654	1.38	1.05E-04	1.70
PS(P-36:5)	+	M+Na	788.4841	0.60	0.050	1.57
PS(P-38:0)	+	M+H	804.6137	3.00	0.027	1.46
**PI**						
PI(34:3)	-	M-H	831.5065	4.37	0.008	1.65
PI(36:4)	-	M-H	857.5220	4.05	0.030	1.26
PI(36:5)	-	M-H	855.5067	4.46	0.001	2.05
PI(38:5)	-	M-H	883.5383	4.67	0.001	1.48
PI(40:6)	-	M-H	909.5539	4.50	0.019	1.38
**Other PL**						
PA(O-42:6)	-	M-H	761.5483	-0.96	0.002	2.44
**Sphingomyelin**						
SM(d32:1)	+	M+H	675.5445	1.44	0.001	1.31
	-	M+FA-H	719.5374	4.40	0.004	1.42
SM(d33:1)	+	M+H, M+Na	689.5603	1.53	0.007	1.36
SM(d34:1)	+	M+H	717.5565	3.26	4.83E-05	1.69
SM(d34:2)	+	M+H, M+Na, M+K	701.5604	1.66	0.006	1.25
SM(d36:1)	-	M+FA-H	775.5991	2.72	0.001	1.56
SM(d36:2)	-	M+FA-H	773.5832	2.41	0.006	1.39
SM(d36:3)	+	M+H	727.5754	0.78	0.040	1.21
SM(d39:1)	+	M+K	811.6058	-4.13	0.049	1.60
SM(d40:2)	-	M-H	783.6424	4.95	0.014	1.37
SM(d42:3)	-	M+FA-H	855.6627	3.73	0.044	1.23
**Sphingolipids**						
Cer(d32:2(2OH))	+	M+H	524.4672	-0.21	0.008	1.53
HexCer (d32:2)	+	M+Na	832.6604	-4.12	0.012	1.40
HexCer(d32:1)	+	M+Na, M+K	834.6776	-2.09	0.000	1.44
HexCer(d34:1(2OH))	-	M+FA-H	760.5558	-3.08	0.029	2.63
HexCer(d41:1)	-	M-H, M+FA-H, M+K-2H	842.6762	4.39	0.006	1.48
HexCer(d42:1)	-	M-H	810.6862	4.10	0.002	1.44
HexCer(d42:2)	-	M-H	808.6657	-1.81	0.042	1.32
GalNAcβ1-4(NeuGcα2-3)Galβ1-4Glcβ-Cer(d42:1)	+	M+H, M+Na	1484.9231	2.12	0.043	1.33

**Diacylglycerols**						
DG(36:4)	+	M+Na, M+K	655.4708	1.49	0.038	1.26
DG(37:4)	-	M-H	629.5174	3.67	0.001	3.53
DG(38:5)	+	M+Na	665.5086	-4.65	0.044	1.22
**Triacylglycerols**						
TG(46:6)	+	M+H	767.6166	-2.43	0.019	2.93
TG(47:3)	+	M+NH4	804.7080	0.57	0.019	Infinity
TG(48:7)	+	M+H	793.6328	-1.64	0.048	2.45
TG(50:9)	+	M+NH4	834.6613	0.85	0.001	1.46
TG(51:8)	+	M+H	833.6636	-2.18	0.007	1.83
TG(53:9)	+	M+H	859.6792	-2.07	0.017	1.68
TG(54:12)	+	M+K	905.6059	0.35	0.041	1.23
TG(55:10)	+	M+K	923.6535	1.06	0.012	44.88
TG(61:4)	+	M+NH4, M+Na	835.6793	0.83	0.040	1.71
TG(66:4)	+	M+NH4, M+Na, M+K	1073.9468	1.99	0.043	3.07
TG(66:5)	+	M+Na, M+K	1071.9288	-0.23	1.18E-04	10.01
TG(68:5)	+	M+Na, M+K	1099.9628	0.66	0.044	2.20

Table ESM 3. Lipid species increased in the plasma of hBace1 knock-in mice compared to WT controls. Shown are specific lipid species, ionisation mode (+ positive or - negative), adducts, m/z values (mass/mass spectrum charge number), mass error, p values obtained from ANOVA analysis and fold-change compared to WT controls. Abbreviations: *PC* phosphatidylcholine (>50% of total phospholipids), *PE* phosphatidylethanolamine, *PI* phosphatidylinositol, *PL* phospholipids and *PS* phosphatidylserine.
