# Supplementary material for: Neuronal human BACE1 knockin induces systemic diabetes in mice
Source: Diabetologia. 2016 May 2;59:1513–23. doi: 10.1007/s00125-016-3960-1 (PMC4901117; doi:10.1007/s00125-016-3960-1)
Supplement: Supplementary file 4 — (PDF 124 kb) [file 125_2016_3960_MOESM4_ESM.pdf]

ESM Table 4

Identification	Ionisation Mode	Adducts	m/z	Mass Error (ppm)	Anova (p)	Fold Change
**Phospholipids**						
**PC and LysoPC**						
PC(14:0)	+	M+H	468.3093	1.83	2.83E-06	2.13
	-	M+FA-H	512.3009	3.28	0.005	1.63
PC(22:1)	+	M+H	578.4187	1.17	0.030	1.15
PC(38:4)	+	M+H	810.6037	3.66	0.023	1.76
PC(42:0)	+	M+K	912.6834	1.79	0.008	1.60
PC(42:4)	+	M+H	866.6642	1.05	0.043	1.19
PC(44:7)	+	M+H	888.6496	2.10	0.018	1.23
**PE and LysoPE**						
PE(16:0)	-	M-H	452.2798	3.29	0.005	1.73
PE(18:0)	+	M+H	482.3251	2.12	0.036	1.40
PE(44:6)	+	M+H	848.6148	-1.90	0.038	1.81
PE(O-38:5)	+	M+H	752.5620	4.18	0.012	1.65
**PS**						
PS(43:0)	-	M+FA-H	934.6719	-3.88	0.017	2.68
**Sphingolipids**						
Cer(d34:1)	-	M-H	536.5063	2.78	0.032	2.32
Cer(d38:1(2OH))	-	M-H	608.5650	4.32	0.022	Infinity
Cer(t38:0)	-	M-H	610.5810	4.99	0.002	1.54
Lactosylceramide (d44:2)	+	M+Na	1022.7442	-3.57	0.033	1.20
NeuAcalpha2-3Galbeta1-4GlcNAcalpha1-3Galbeta1-4GlcNAcbeta1-3Galbeta1-4Glcbeta-Cer(d42:1))	-	M-H	1992.0813	0.73	0.017	2.44
NeuGcalpha2-3Galbeta1-3GalNAcbeta1-4(NeuGcalpha2-3)Galbeta1-4Glcbeta-Cer(d46:2)	+	M+H	1952.0497	1.15	0.048	2.29
**Diacylglycerols**						
DG(34:3)	+	M+Na	613.4792	-1.70	0.046	3.61
**Triacylglycerols**						
TG(56:7)	+	M+NH4	922.7900	4.60	0.015	1.19
TG(58:12)	+	M+NH4	954.7557	1.28	0.022	1.31
TG(62:5)	+	M+NH4	1024.9243	-2.34	0.048	5.34

Table ESM 4. Lipid species decreased in the plasma of hBace1 knock-in mice compared to WT controls. Shown are specific lipid species, ionisation mode (+ positive or - negative), adducts, m/z values (mass/mass spectrum charge number), mass error, p values obtained from ANOVA analysis and fold-change compared to WT controls. Abbreviations: *PC* phosphatidylcholine (>50% of total phospholipids), *PE* phosphatidylethanolamine, *PI* phosphatidylinositol, *PL* phospholipids and *PS* phosphatidylserine.
